# Supplementary material for: Identification of photoperiod-induced specific miRNAs in the adrenal glands of Sunite sheep (Ovis aries)
Source: Front Vet Sci. 2022 Jul 22;9:888207. doi: 10.3389/fvets.2022.888207 (PMC9354845; doi:10.3389/fvets.2022.888207)
Supplement: Supplementary file 1 [file Data_Sheet_1.ZIP › Supplementary file/miRDeep2 file.zip!/miRDeep2 file/Know_Structure/SPLP42A2/oar-mir-496.pdf]

miRBase precursor : oar-mir-496
Total read count : 13
oar-miR-496-5p read count : 2
oar-miR-496-3p read count : 11
remaining reads : 0

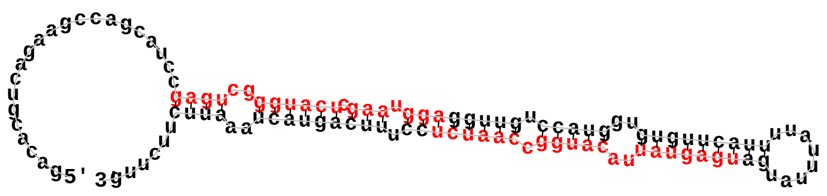

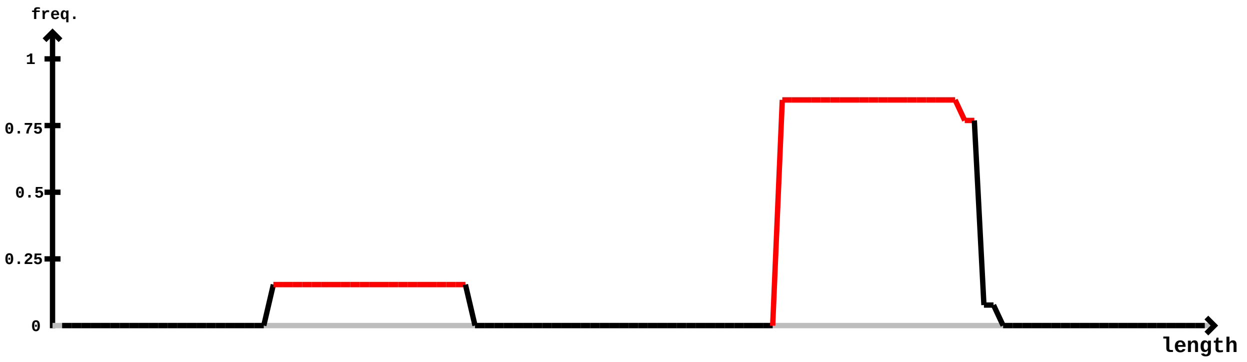


```
                                       oar-miR-496-5p                                          oar-miR-496-3p
5'- gacacgucagaagccagcauccgagucggguacucgaauggagguuguccaugguguguucauuuuauuuaugaugaguauuacauggccaaucuccuuucaguacuaaauucuucuug   -3'   exp
    ......................((((..((((((.(((.((((((((.(((((..(((((((((........)))))))))..))))).)))))))).)))))))))..)))).......   reads   mm   sam
    .....................cgagucggguacucgaaugga.............................................................................   2       0    sec
    ..........................................................................ugaguauuacauggccaau..........................   1       0    sec
    ..........................................................................ugaguauuacauggccaaucu........................   9       0    sec
    ..........................................................................ugaguauuacauggccaaucucc......................   1       0    sec
```
